# 3D Traction Force Microscopy in Biological Gels: From Single Cells to Multicellular Spheroids

**DOI:** 10.1146/annurev-bioeng-103122-031130

**Published:** 2024-06-20

**Authors:** Brian C.H. Cheung, Rana J. Abbed, Mingming Wu, Susan E. Leggett

**Affiliations:** 1 Department of Biological and Environmental Engineering, Cornell University, Ithaca, New York, USA; 2 Department of Bioengineering, University of Illinois Urbana-Champaign, Urbana, Illinois, USA; 3 Cancer Center at Illinois, University of Illinois Urbana-Champaign, Urbana, Illinois, USA

**Keywords:** traction force microscopy, cell force measurements, extracellular matrix deformation, biological gels, 3D models, mechanobiology

## Abstract

Cell traction force plays a critical role in directing cellular functions, such as proliferation, migration, and differentiation. Current understanding of cell traction force is largely derived from 2D measurements where cells are plated on 2D substrates. However, 2D measurements do not recapitulate a vital aspect of living systems; that is, cells actively remodel their surrounding extracellular matrix (ECM), and the remodeled ECM, in return, can have a profound impact on cell phenotype and traction force generation. This reciprocal adaptivity of living systems is encoded in the material properties of biological gels. In this review, we summarize recent progress in measuring cell traction force for cells embedded within 3D biological gels, with an emphasis on cell–ECM cross talk. We also provide perspectives on tools and techniques that could be adapted to measure cell traction force in complex biochemical and biophysical environments.

## INTRODUCTION

The complex extracellular milieu presents diverse biochemical, electrical, topographic, and mechanical cues to cells and tissues that govern all stages of life from birth to death. The ability of cells to sense and respond to their surrounding microenvironment is critical for the maintenance of physiological homeostasis. Accordingly, pathological states may arise in response to aberrations in the cellular microenvironment and the dysregulation of cell machinery that interfere with sensory transduction. Groundbreaking research in the 1950s radically changed our understanding of the mechanics of cellular function. Notably, Huxley & Hanson identified the location and arrangement of actin and myosin in striated muscle (1). These findings ultimately led to the development of Huxley’s sliding filament theory and the swinging crossbridge model characterizing the movement of myosin along actin filaments (2). Huxley & Hanson’s work inspired the study of intracellular machinery that enables cellular force generation, launching the rapidly evolving field of mechanobiology.

It is now well accepted that cells sense and respond to mechanical cues within the surrounding extracellular matrix (ECM), which directs cellular processes such as migration, differentiation, and the regulation of gene expression (3–8). For example, mechanical cues govern the determination of stem cell fate by matrix stiffness (9), depolarization of inner hair cells via transduction of fluid stress (10), guided migration of neural crest cells toward stiffer environments during embryonic development (11), and enzymatic degradation of a stiff microenvironment to generate micropaths for metastasis (12). While the mechanics of the microenvironment can influence cellular behaviors, cells can also exert forces on the matrix to guide protrusions by altering the microarchitecture and fiber orientation of ECMs (13, 14). In breast cancer, tumor cells are known to exert forces on their surrounding tissue and reorganize the architecture of ECMs. The heterogeneous and aligned collagen matrices, in return, promote tumor invasion into the tissue and are important clinical biomarkers for the state of malignancy in tumors (15). As such, cell–ECM reciprocal interactions are important for both homeostatic and pathologic processes.

The mechanical properties of biological gels are unique in that they can facilitate reciprocal cell–ECM interactions. For example, a much-studied biological gel is type I collagen, which is a fibrous network that provides mechanical support for tissues and is understood on the basis of semiflexible polymer physics. A critically important feature of biological gels is that they are adaptable to cells in both the spatial and temporal scales that enhance tissue functions. Biological gels strain stiffen when stretched in space, a characteristic that facilitates cell–ECM and cell-cell communication (16) and is important for tissue durability under tension (17–19). Biological gels are also viscoelastic, a quality that allows the cells to tune the timescale of the gel to promote their functions, including proliferation and migration (20). The rapid establishment of synthetic gels has enabled distinct features of biological gels to be mimicked and tuned (21–24). While synthetic materials can be customized to tease out the distinct contributions of mechanical or biochemical cues to cellular function, natively derived materials better recapitulate the physiological state. Studies of cell function in both synthetic and natively derived gels can provide complimentary information on cell–ECM interactions, provide guidelines for future development of synthetic gels for applications in tissue engineering, and enable drug testing and disease treatments (25–27). However, the study of cell–ECM interactions and traction force in complex microenvironments has been limited to date, hindering insights into the governing role of mechanical forces across all stages of life, including embryogenesis, growth, development, aging, and disease. Here, we provide a detailed review of the recent technological advances that enable the measurement of cell traction forces in biological gels, with a special focus on the biological insights gained from elucidating cell–ECM cross talk in tumor models. For a tutorial on cell traction force measurements, readers are referred to Reference 28. For information on comprehensive methods in cell traction force measurement methods, readers are referred to References 29–31. For a discussion on microfabricated methods to measure cell and tissue force, readers are referred to Reference 32.

## WHAT IS CELL TRACTION FORCE?

**Figure 1:**
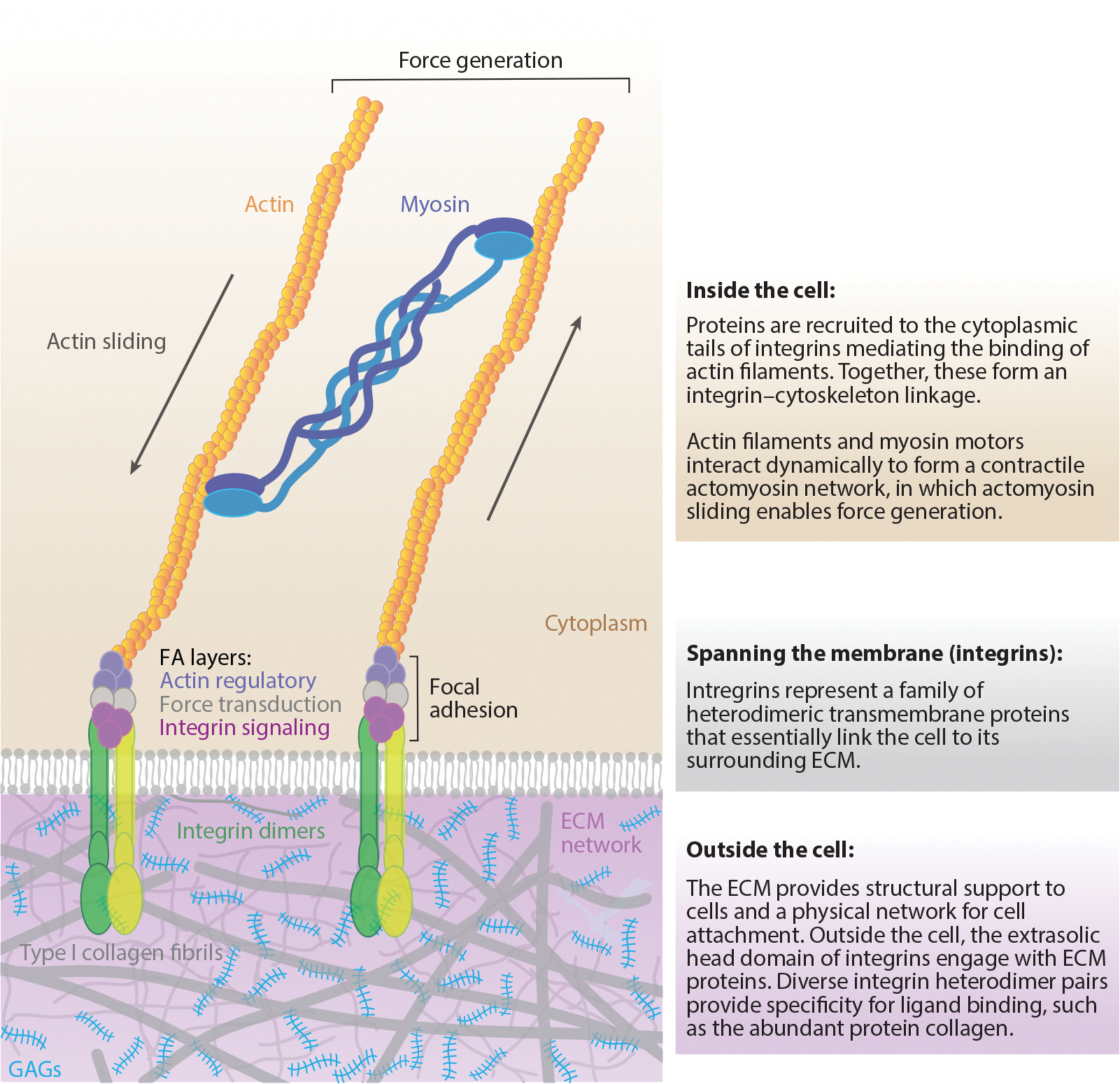
Definition of cell traction force. Cells generate and transmit mechanical forces on the surrounding ECM, which are termed traction forces. Cell traction force is enabled by the orchestration of intracellular and extracellular machinery, which is critically regulated by the actomyosin network and integrins. Figure is not drawn to scale. Abbreviations: ECM, extracellular matrix; FA, focal adhesion; GAG, glycosaminoglycan.

The generation of cell traction force requires two critical components: (*a*) the intracellular machinery that provides the energy source of the force, that is, the actin–myosin network, and (*b*) the physical cell–ECM linkage between cell surface adhesion receptors and the ligands within the ECM (see Figure 1).

### The Cytoskeleton: A Complex Network Within the Cell for Supporting Cell Structure and Traction Force

The biological mechanism enabling cell traction force involves both the coordination of intracellular machinery and the formation of physical cell–ECM linkages. The cell cytoskeleton is a dynamic, self-organizing network primarily composed of actin, microtubules, and intermediate filaments that critically regulate cell form, function, and physical forces. Notably, actin is the most abundant component of the cytoskeleton for most eukaryotic cells. The assembly of globular actin monomers (G-actin) generates filamentous actin (F-actin), which provides mechanical support and the structural framework to regulate cell shape and intracellular organization (33). The rapid polymerization and depolymerization of actin support the highly dynamic nature of the cytoskeleton that is required to coordinate cell migration, adhesion, and contractility. Actin polymerization at the leading edge of the cell generates protrusions, including filopodia and lamellipodia, which interact with the ECM to generate protrusive and contractile traction forces (34). As Huxley & Hanson recognized early on, actin and myosin are major molecular players that cooperate to regulate cellular behavior (2). Specifically, interactions between F-actin and the molecular motor protein, myosin, produce cell tension through the contraction of stress fibers. Microtubules play a supporting role in cytoskeletal organization and traction force generation by forming dynamic polarized tracks along which dynein and kinesin motor proteins can transport cargo to the cell’s leading edge. For instance, Seetharaman et al. (35) demonstrated that microtubule acetylation enhanced the magnitude of traction forces exerted by human umbilical vein endothelial cells and astrocytes. Similarly, fibrous proteins that make up intermediate filaments, such as keratins and vimentin, aid in the stabilization and distribution of traction forces generated by actin and microtubules (36). Overall, the cytoskeleton enables the generation of mechanical forces that are exerted on neighboring cells and the surrounding ECM (Figure 1). These traction forces can be propagated across long ranges, influencing cell behavior and the physical properties of the tissue microenvironment.

### Cell–ECM Interactions in Traction Force Generation

Cell attachment to the surrounding ECM is fundamentally regulated by surface adhesion receptors such as integrins. A wide array of distinct alpha and beta integrin subunits assemble into heterodimers, composing a superfamily of cell adhesion proteins that uniquely bind to specific sites on ECM proteins and aid in mechanotransduction (37). This ligand specificity can be generally broken down into five main categories of integrin binding: collagen receptors, laminin receptors, RGD (arginine–glycine–aspartic acid) receptors, non-RGD receptors, and leukocyte-specific receptors. For instance, integrins α_6_β_4_ and α_3_β_1_ are critical for epithelial cell binding to type IV collagen and laminin in the basement membrane, respectively. Conversely, cell interactions with the major constituents of the interstitial matrix, type I collagen and fibronectin, are regulated by integrins α_5_β_1_ and α_1/2_β_1_, respectively (38). Importantly, integrin clustering generates specialized adhesion sites, or focal adhesions, which are highly dynamic multiprotein structures primarily composed of integrins, cytoskeletal proteins, and signaling molecules (39). Focal adhesion proteins, such as talin, vinculin, and paxillin, form complexes that connect integrins and actin together (40). Subsequently, signaling molecules (e.g., focal adhesion kinase, paxillin, and Src family kinases) are recruited to mediate downstream signaling (41). Actin binding proteins, such as α-actinin and vasodilator-stimulated phosphoprotein, serve as actin regulatory proteins that, when bound, enhance actin stress fiber attachment and traction force generation (42). Critically, these dynamic complexes form mechanical bridges between the intracellular cytoskeletal network and the ECM, known as cell–matrix adhesions, that enable mechanosensation, mechanotransduction, and force generation (see Figure 1).

The relative abundance of cytoskeletal proteins differs across cell types, and, thus, traction force generation can vary dramatically. For instance, cell types with prominent actin cytoskeletons, such as fibroblasts, skeletal muscle, and smooth muscle cells, are highly contractile and generate substantial traction forces. Moreover, cell plasticity can give rise to altered morphological states. For example, the epithelial-to-mesenchymal transition is associated with dramatic cellular elongation and an increase in vimentin expression (43). Correspondingly, F-actin reorganization and altered vimentin expression enhance the contractility of cells undergoing the epithelial-to-mesenchymal transition (44). Overall, cell traction force generation is intricately related to cytoskeletal dynamics, as well as the composition and physical properties of the surrounding ECM. However, while 3D cell migration has been well studied (45), our understanding of cytoskeletal dynamics and matrix interactions in 3D microenvironments remains nascent relevant to 2D. As such, additional studies are needed to examine cell traction force in a more physiologically relevant 3D context, which may be addressed by embedding cells in biological gels.

## MECHANICAL PROPERTIES OF BIOLOGICAL GELS

Biological gels are biopolymers that provide structural support in soft living materials. A unique mechanical property of biological gels is their modifiable nature, in that the architecture and biochemical composition change in the presence of cells. Here, we focus on methods used to characterize and model mechanical properties of biological gels. Examples are given for studies of type I collagen, an abundant ECM protein that is responsible for mechanical support of tissue and is often dysregulated in disease.

### Measuring Mechanical Properties of Biological Gels

**Figure 2:**
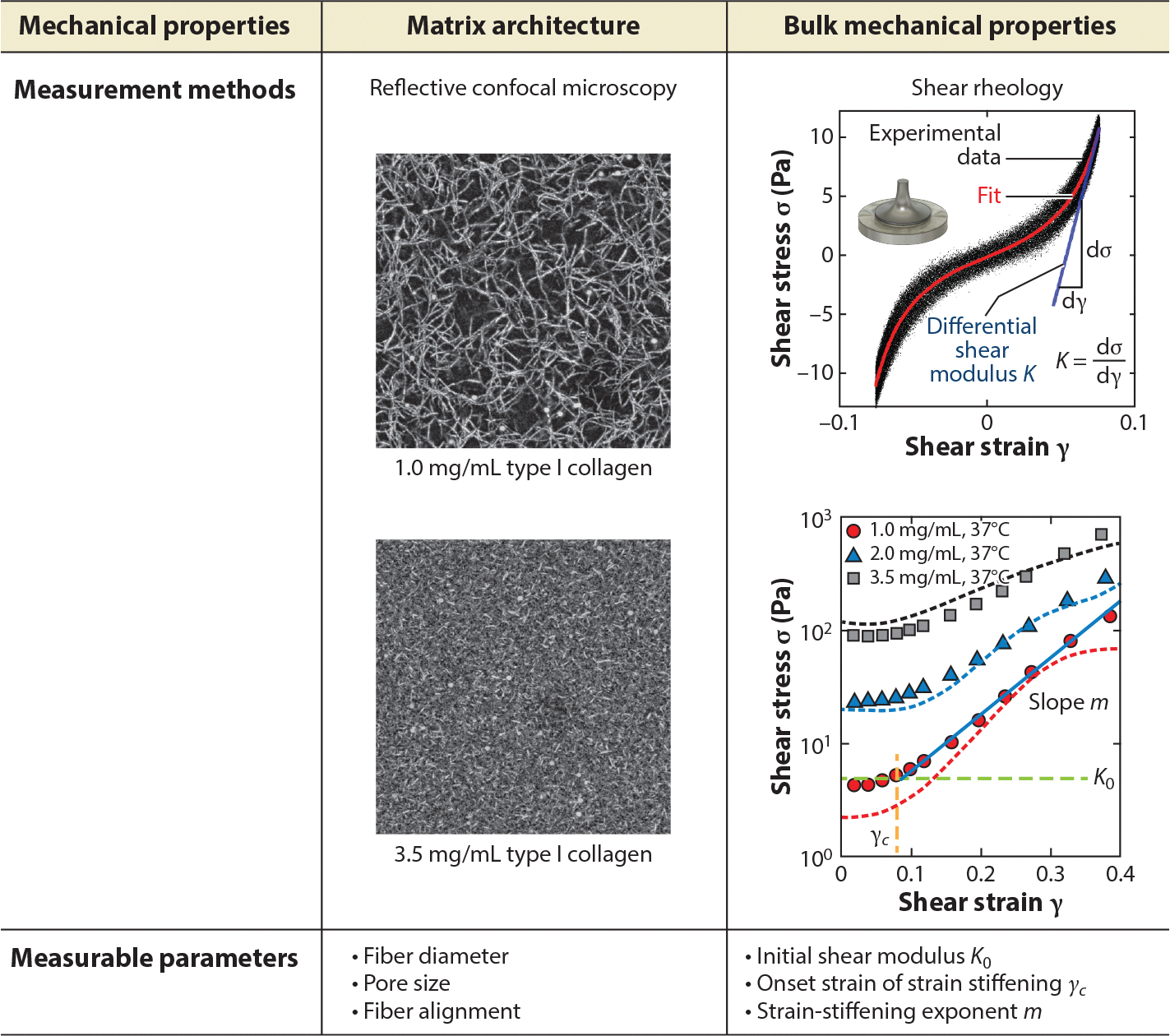
Characterization of the mechanical properties of extracellular matrices (ECMs). Mechanics of ECMs are typically described by their matrix architecture and bulk mechanical properties. Here, we show the common techniques used in quantifying the mechanics of ECMs and the parameters generated from these techniques. Reflectance confocal images show the representative fiber networks of biological gels with distinct collagen protein concentrations, as indicated (98 × 98 μm). Parallel rheometer results show shear modulus of collagen at three different collagen concentrations: 1.0, 2.0, and 3.5 mg/mL. Images adapted from Reference 17. Graphs adapted from Reference 61.

Two important mechanical properties of biological gels critical for cell–ECM interactions are ECM architecture and bulk mechanics. Bulk mechanical properties are typically characterized using an oscillatory rheometer (46) (see Figure 2). Here, a parallel plate oscillatory rheometer is used to first obtain the differential modulus of the gel, and then the strain stress relation can be plotted for type I collagen of various concentrations. Interestingly, all biological gels including collagen, vimentin, actin, and fibrin display strain-stiffening properties, as presented in the pioneering work by Storm et al. (47). These properties are encoded in tissue function such that cardiac tissue, for example, can sustain large deformation without being torn apart. In addition, cells can optimize their way of communication using the strain-stiffening property. It has been found that the propensity of strain stiffening promotes long-range cell–cell communication (17, 48). Equally important are the viscoelasticity and plasticity of biological gels, which provide timescales essential for regulating cell adhesion, spread, and migration (20, 49). A rheometer measures the bulk mechanics of an ECM. A number of other tools have been developed to measure local properties of the ECM, including active microrheology techniques that involve the manipulation of probe particles through optical forces, such as optical tweezers (50) and light radiation pressure force (51). The latter technique is also capable of acquiring a large volume of images (>400 z slices) in under 10 min, a result that is unprecedented in conventional confocal microscopy (51).

Largely owing to the introduction of advanced microscopy and optical techniques, it is now increasingly clear that in addition to bulk mechanical properties, the architecture of biological gels is critical to cell function. In the tumor microenvironment, regions of healthy tissue often display a homogeneous and isotropic fiber network architecture, while diseased tissue shows heterogeneous and aligned network architecture (52, 53). The best-studied biological gel is type I collagen, of which the pore size and fiber diameter are two important parameters for regulating cell function, in particular migration (see Figure 2). It is known that aligned collagen promotes tumor cell invasion and is a poor prognosis of tumor malignancy (54). A small pore size, when comparable to cell nuclear size, is the limiting factor for cell migration within 3D ECM and is known to promote proteolytic activities of tumor cells to create microsized channels within the ECM for migration. The bulk mechanics of the biological gels are directly correlated with their fiber network architecture. This is beautifully illustrated in theoretical work by a number of groups (see, e.g., 50, 55), in that strain stiffening is a result of fiber alignment, and the onset of the strain stiffening as well as the level of strain stiffening is closely related to the pore size and fiber diameter of the fiber network.

### Tuning Mechanical Properties of Biological Gels

**Figure 3:**
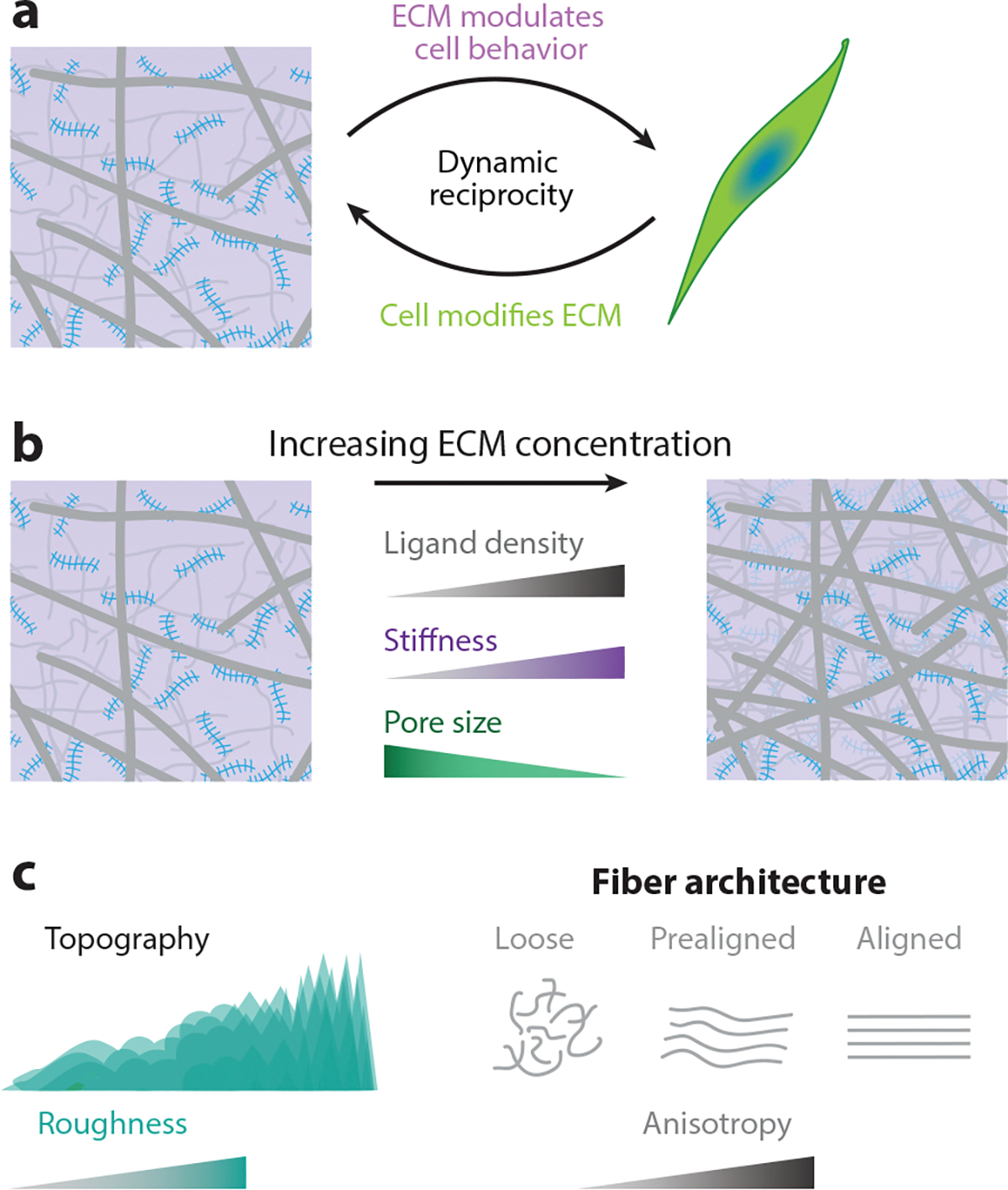
Biological gels facilitate mechanical cross talk between cells and ECMs. (*a*) Reciprocal interactions between cells and the surrounding extracellular matrix (ECM) dictate cell behavior and ECM changes, occurring as a continuous feedback loop known as dynamic reciprocity. (*b*) Generally, as ligand density within the ECM increases, matrix stiffness increases, while pore size decreases. (*c*) ECM remodeling can produce dramatic spatiotemporal changes in matrix topography across length scales, such as the progressive transition to ECM anisotropy.

Mechanical cross talk between cells and the altered ECM facilitates an adaptive cell response, for which altered cell behavior can drive further ECM changes. In their pioneering work, Bissell et al. (56) coined the term dynamic reciprocity to describe this bidirectional feedback loop, which remains a guiding principle for the field of mechanobiology (57) (Figure 3a). Biological gels with defined mechanical cues can be employed to better understand this cell–ECM cross talk in a physiologically relevant microenvironment. A simple method to tune the mechanical properties of biological gels is to vary the concentration of chief structural ECM components within the hydrogel (e.g., type I collagen) (Figure 3b). It is well known that increasing collagen concentration results in a matrix with increased stiffness and ligand density but decreased pore size (58) (also see Figure 2). These changes may also be associated with alterations in local topography and matrix anisotropy (Figure 3c). In addition to modulating integral components in the ECM, one can also modulate ECM mechanical properties via cross-linking. Enzymatic lysyl oxidase–mediated cross-linking increases collagen stiffness and can promote tumor malignancy (59). Nonenzymatic ribose cross-linking in collagen has also been found to increase collagen gel stiffness. For instance, the addition of ribose at 100 mM was shown to increase collagen stiffness by several orders of magnitude. This cross-linking promotes tumor invasion, which presents a potential mechanic link between diabetics and cancer (60).

A second example of an essential ECM component is the glycosaminoglycan (GAG) family of linear polysaccharides, which form interpenetrating networks in the collagen matrix. Critically, while collagen fiber networks within biological gels sustain tension within a tissue, GAGs sustain compression. GAGs play important roles in several homeostatic and pathologic processes including cardiac development, fibrosis, and solid tumor progression (48). Recent work from the Shenoy lab (48) showed that GAGs modulate ECM architecture and decrease cells’ propensity to transmit forces in space, thus reducing cell–cell interactions. It has also been shown that cell traction force is reduced in the presence of GAG (61). The last example is Matrigel^®^, a raw biological material derived from Engelbreth–Holm–Swarm mouse sarcoma, which has been commonly used as a basement membrane mimic in organoid culture. The viscoelastic properties of Matrigel have been recently characterized using optical tweezer–based microrheology, which demonstrated that the matrix becomes more elastic as a function of increasing polymer concentration after gelation (62).

Nonmammalian biological gels have also been employed to expand the repertoire of biological hydrogels for cell–matrix studies and to create materials with new mechanical properties. Pioneering work by the Kaplan lab (63) has established biocompatible and biodegradable silk fibroin (SF) hydrogels prepared from silk produced by *Bombyx mori* silkworm cocoons. Recent advances in physical and chemical cross-linking methods have enabled the generation of SF hydrogels with a wide range of mechanical properties (e.g., porosity, mechanical strength, and swelling) (64). Composite hydrogels can further expand the range of mechanical microenvironments with which to study cell–matrix interactions. For instance, by increasing the concentration of SF within SF–type I collagen hydrogels, the Wong group (65) demonstrated that matrix stiffness and architecture could be controlled independently of collagen concentration, which resulted in the mesenchymal-to-amoeboid transition of breast cancer cells. Like SF, the polysaccharide alginate lacks integrin binding sites for cell adhesion (66). This feature has been leveraged to strategically engineer alginate hydrogels with coupled RGD sites and tunable viscoelasticity (21, 67). Overall, biological gels with defined mechanical properties can be leveraged to investigate cell force generation within a physiologically relevant context.

## MEASURING SINGLE-CELL TRACTION FORCES

**Figure 4:**
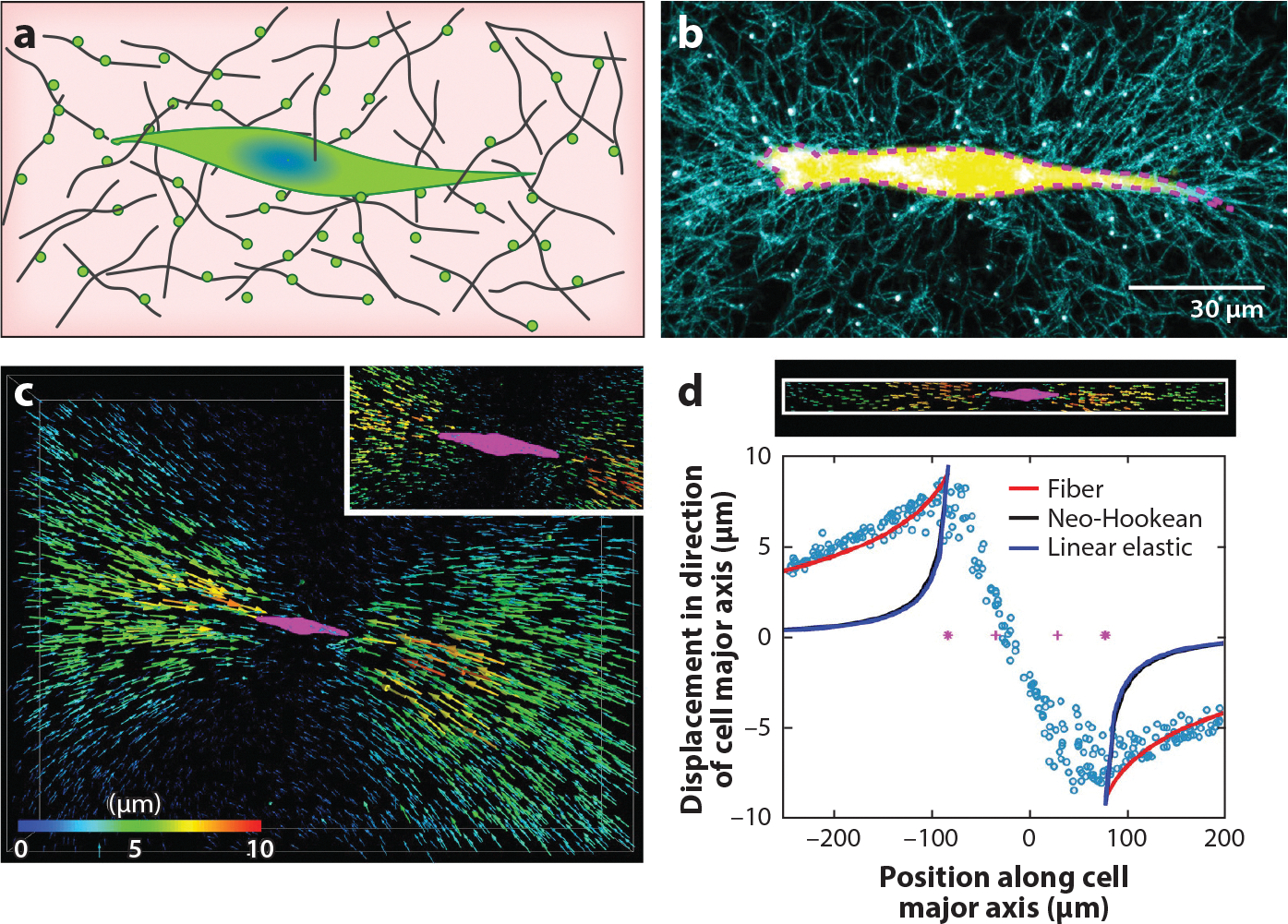
Single-cell traction force measurement in 3D. (*a*) Illustration of a cell embedded within a biological gel for traction force microscopy. Black lines are the semiflexible polymer network supporting the cells; green dots are the fluorescent marker beads covalently bonded to the fibers to assist in visualization of matrix deformation. (*b*) An image of a single cell (*yellow*) embedded within a type I collagen matrix. The type I collagen fibers and fluorescent beads were imaged using confocal microscopy. (*c*) Matrix deformation field due to single-cell traction, revealed by a particle tracking technique. (*d*) Fitting of bead displacement along the cell’s major axis using different material models. Panels *b*–*d* adapted from Reference 17.

Measurement techniques for single-cell traction forces have evolved rapidly in the past 20 years, from early measurements of cell traction force on 2D substrates to recent work of cells embedded within biological gels (Table 1). With a few exceptions, the basic process behind cell traction force measurements is to first quantify the material deformation caused by cell traction force and to then use the deformation and material property to calculate the cell-generated traction force (Figure 4). When the material is linear elastic [e.g., micropillars or polyacrylamide (PA) gels], the computation from material deformation to cell traction force is straightforward. On the other hand, determining cell traction force with nonlinear elastic materials poses a challenge, especially in the case of biological gels that undergo cell-driven matrix remodeling. In this section, we focus on techniques and advancements in visualizing ECM deformation and the computational models used in translating matrix deformation into cell traction force for individual cells embedded in biological gels.

### Particle Tracking–Based Traction Force Microscopy

**Figure 5:**
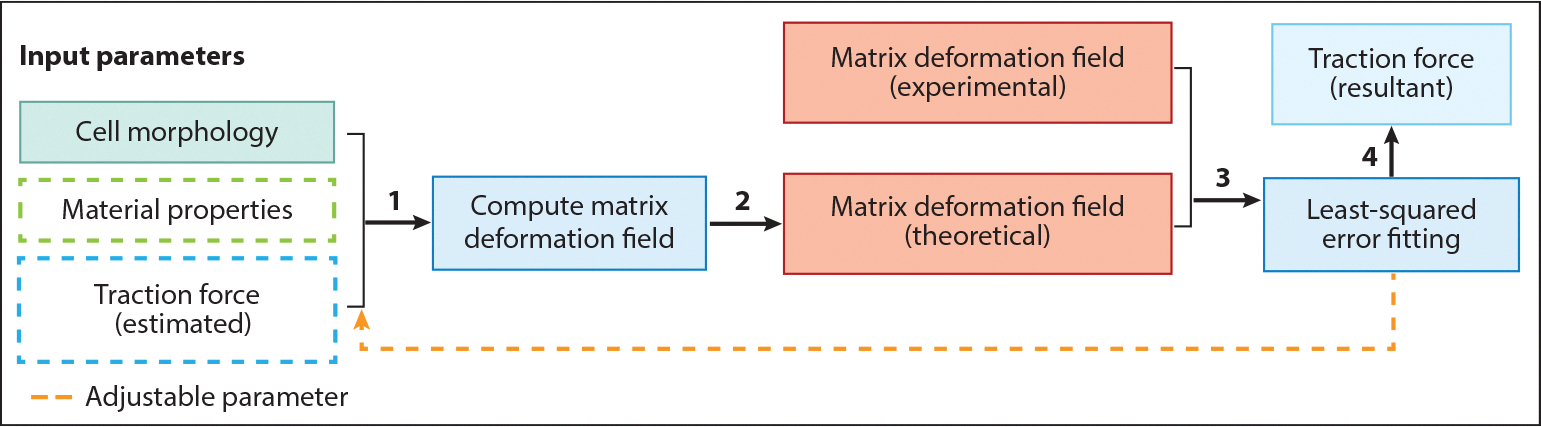
Flowchart of traction force computation in 3D. Step 1: Obtain cell morphology and extracellular matrix (ECM) properties from the experiment and make an initial estimate of the traction force. Step 2: Compute the ECM deformation field using the constitutive material model. Step 3: Use least-squared error fitting to match the computed with the experimentally measured matrix deformation field. Step 4: Translate the result of the fit into cell traction force.

Traction force microscopy (TFM) is a single-cell force measurement technique enabled by harnessing a cell’s ability to pull on a substrate/ECM that it adheres to and the known material properties of the substrate/ECM. The central idea for all of the particle tracking–based TFM techniques is to first visualize the matrix deformation caused by cell traction force using embedded fiducial markers and to then translate the deformation field into a force field using the known material properties (see Figure 5).

#### Matrix deformation measurement in 3D.

To fully recapitulate cell traction in 3D, our labs and others have embedded cells in 3D biological gels, closely mimicking their native environments (17, 68–73). Matrix deformation caused by the release of the traction force was measured using two images, an image of the ECM when a cell exerts force onto it, and a reference image when cell force is relaxed. The 3D ECM coordinates are identified by the embedded fluorescent beads typically covalently bonded to the matrix. Epifluorescence experiments are commonly performed using z stacks or confocal microscopes (17, 74). The first challenge of 3D single-particle tracking is to locate bead positions in a 3D volume. When using an epifluorescence microscope, the challenge is to increase the resolution in the vertical position of the beads, as this is limited by the number of z-slice images taken. Point spread function–based methods have been developed to circumvent this problem and are able to achieve subpixel accuracy (17, 74–77). For confocal imaging, the in-plane and z resolution are determined by the numeric aperture of the objective lens and pinhole, respectively. Recent developments in confocal microscopy have significantly improved our ability to follow cell traction force. To push the spatial resolution limit, super-resolution imaging techniques such as 3D structured illumination microscopy (SIM) (78) and single-molecule localization microscopy (79) provide resolutions down to a few tens of nanometers, at the cost of a longer image acquisition time.

To compute the ECM deformation using both the image when cell traction force is active and the reference image, computation algorithms have been developed to circumvent the challenges posed in 3D. Early endeavors including particle-tracking velocimetry using nearest neighbor search (80–82) have enabled robust mapping of displacement fields in 2D. These methods have limited success in 3D (83) due to the relatively low spatial resolution of images taken in 3D. Feature vector (FV)-based algorithms (70, 84) have been developed and used successfully in 3D TFM. Instead of connecting the particle to its closest neighbor in the previous frame, FVs are drawn by connecting each bead to multiple (e.g., three) nearest neighbors to generate a unique feature vector. Comparisons are made using these FVs to identify the best match, and displacement is calculated. While FV-based methods have made 3D particle tracking possible, they are often limited to small deformation (85). To address large deformation, the Franck lab (74, 85) has developed a rapid topology-based particle tracking (TPT) algorithm to enhance tracking accuracy at a low computational cost. In TPT, particle tracking is done in a three-step process. First, particles are localized using the radial symmetry method. Then, FVs are generated by linking particles within concentric shells of equal volume. Each shell is divided into eight octants, and the relative positions of the neighboring particles are labeled in each octant. Since particles tend to remain within the same octant, the TPT algorithm can handle large deformation by allowing sufficient freedom for particles to rearrange. Lastly, an iterative deformation warping scheme is used to track particle displacements. TPT has been implemented in the mechanophenotyping of multicellular clusters on the basis of how they deform the matrix (86). Besides FV-based methods, deformation mapping can also be performed using correlation-based methods, such as particle image velocimetry (87, 88), digital image correlation (89), and fast iterative digital volume correlation algorithms (69), which analyze the entire 3D volume. These methods are capable of tracking large deformation fields but do not work well in complex deformation that involves small-scale features, due to their averaging nature (90).

#### Computational algorithm of traction force field using deformation field and extracellular matrix material properties.

To calculate cell traction force using the matrix deformation field and material properties, a number of computation algorithms have been developed. The key challenge is to understand and develop a constitutive model that describes the material properties of biological gels. This is further complicated by the fact that the material properties are modulated by the cell traction force. Here, we present some of the major techniques used in calculating traction force in biological gels.

##### Green’s function–based force computation.

In a linear elastic material, the strain field of a point force in an infinite space (i.e., the Green’s function) is known analytically. Given the experimentally measured displacement field, the cell traction force can be inversely inferred from the convolution integral for the displacement (91). The advantage of this method is that the computation is straightforward. The limitations of this technique are that (*a*) it is largely limited to linear elastic materials, as we now know that all biological materials are nonlinear elastic (47), and (*b*) the boundary conditions are difficult to implement. Noise at the boundary can lead to large deviations in the reconstructed traction field. To reduce artifacts from experimental noise when solving the inverse problem through optimization, regularization is often implemented. However, this technique is difficult to implement and is computationally expensive (92). The first 3D TFM method was pioneered by Legant et al. (70), where the cell traction of NIH 3T3 mouse fibroblasts encapsulated in poly(ethylene glycol) hydrogels was calculated by constructing a discretized Green’s function using finite element analysis. While the fundamental assumptions behind the calculations were matrix linearity and isotropy, this seminal work revealed in 3D that cells exert inward tractions through protrusions. The major limitation of this work is that it was done in a linear elastic material, which does not capture the important fibrous nonlinear elasticity of biological gels. A second issue is that it can take hours to analyze one single dataset (70). To reduce computational time, the resolution must be sacrificed by simplifying the finite element mesh. Alternatively, the traction field can also be solved using other nonregularized methods such as Fourier transform traction cytometry (93). Paradoxically, this method is subject to experimental noise due to the lack of regularization (88).

##### Traction force computation based on constitutive model for biopolymer network.

To compute cell traction force in biological gels, constitutive models have been developed for biopolymer networks and used successfully in computing cell traction force (48, 73, 94). The common feature of biopolymer network models is their inclusion of ECM architecture, as well as their bulk mechanics. This is important, as we now know that cells respond sensitively to ECM pore size, fiber diameter, and gel stiffnesses.

In a seminal work by Steinwachs et al. (73), a semiaffine biopolymer network model was developed to compute the cell traction force using ECM deformation due to single-cell traction force generation within a collagen gel. In this model, collagen gels exhibit three different mechanical phases (buckling, linear, and strain-stiffening) under an applied strain. To address cell-generated forces with complex boundaries and fine protrusions, finite element methods (FEMs) are used to enable the local reconstruction of force with a given cell boundary and deformation field (95, 96). This model has been used successfully to illustrate the roles of collagen concentration in cell traction force generation. Recently, the model has been implemented in calculating the traction of tumor spheroids (97) and immune cells (98). We note that open-source contributions from both the Fabry group (SAENOPY, in Python) (98) and the Van Oosterwyck group (TFMLAB, in MATLAB) (95) have made force calculations easily accessible. While SAENOPY, a 3D TFM solver, allows force calculations in nonlinear materials with a requirement of obtaining displacement data prior to solving, TFMLAB provides image processing routines as well as force calculation modules for both novice and experienced users. TFMLAB, however, supports only linearly elastic materials at the moment.

A fibrous nonlinear elastic material model has been developed and implemented for 3D cell traction force calculation that accounts for the cross talk between cells and the ECM. It is now well accepted that a critical component of cell–ECM interaction is the mechanical cross talk. Cells pull and align the ECM, and the remodeled ECM, in return, influences cell traction force generation (20, 47). A 3D TFM computation algorithm using the fibrous nonlinear elastic material model, jointly developed in the Shenoy and Wu labs, successfully recapitulated the cell–ECM cross talk (17). Here, cells are embedded in a 3D collagen matrix for TFM experiments (Figure 4a,b). The traction force–induced matrix deformation field is measured using two images taken before and after the cell is relaxed (Figure 4c,d). The traction force is computed using the fibrous nonlinear elastic material model and the ECM deformation field. As can be clearly seen in Figure 4b, the ECM is aligned due to cell traction force, which leads to the anisotropic nature of the ECM. The key feature of the fibrous nonlinear elastic material model is that the overall free energy density of the fibrous network under load is contributed by two sets of fibers—isotropic and aligned fibers, *W* = *W*_*f*_ + *W*_*i*_. Here, *W*_*f*_ is the free energy of the aligned collagen, and *W*_*i*_ is the free energy of the isotropic collagen. Without any mechanical load, fibers are randomly oriented. In this state, the mechanical property of the matrix is described by the neo-Hookean hyperelastic model. When a load is applied to the matrix, fibers become aligned in the direction of load and the matrix stiffens, giving rise to the nonlinear stress–strain behavior. The level of ECM alignment is reflected in the relative contribution of these two terms, and the equilibrium state is reached by minimizing the total free energy. This constitutive model provides a straightforward platform to capture the cell–ECM interaction and has been successfully implemented to include contributions from hyaluronic acid in the collagen network (48). A step-by-step flowchart for computing cell traction force using the fibrous nonlinear elastic material model is presented in Figure 5. The first step is to collect input parameters, including the cell shape, the material properties measured by the rheometer (the initial differential shear modulus *K*_0_, the onset of strain stiffening γ_*c*_, and the strain stiffening slope *m*; see Figure 5), and the estimated traction force. The ECM deformation computing module (e.g., an FEM solver) takes these three input parameters and calculates a displacement field using the constitutive fibrous nonlinear elastic material model (17). A second key computing module is a least-squared fitting optimization program (see Figure 5). It takes the computed ECM deformation field and matches it to the experimentally measured ECM deformation field. This optimization program uses the input estimated cell traction force and material properties as adjustable parameters, and the outcome of the fit is the measured cell traction force. We note that the cell–ECM cross talk is reflected in the fitting process, where the cell traction force and the ECM properties are both adjustable parameters. The altered ECM properties are also the outcome of the fitting process, which recapitulates the fact that cells remodel the ECM.

### Deep Learning–Based Traction Force Microscopy

Recent developments of neural network–based computation have been used to develop a more efficient and robust algorithm for translating matrix deformation into cell traction force. A major challenge of TFM is solving the ill-posed inverse problem of translating the ECM displacement field into force (28, 70). To accurately calculate force, one can first assume a traction force exerted by the cell and then compute a deformation field and compare it with the experimentally measured deformation field. This process is repeated until the computed deformation field matches with the experimental data. This process is computationally expensive and often does not converge. Recently, the rapid advancement of neural network–based deep learning techniques has enabled the prediction of cell traction forces on the basis of the images of the ECM taken before and after cell traction force relaxation (99–102). While deep learning–based methods were started in 2D (99, 100), Duan & Huang (101) developed a cellular force–deep convolution neural network (CF-DCNN) to reconstruct traction forces in 3D. In this work, datasets were generated from single cells modeled as ellipsoids surrounded by a hydrogel. The training process was done with supervision, where the theoretical displacement fields prescribed in simulations were set as labels to compare against the predicted results. Compared with the conventional 3D TFM method, CF-DCNN can achieve a comparable accuracy while reducing computational time by two orders of magnitude [~206 s–12.29 h (3D TFM) versus ~22.58 s (CF-DCNN) when processing an image with a size of 512 × 512 × 160 voxels (101)]. Overall, deep learning has offered an alternative approach to accurately predict and assess cell-generated forces with less experimental time needed for collecting and processing real-life data.

## TOWARD ADVANCED MEASUREMENTS OF TRACTION FORCES IN COMPLEX TISSUES

The elucidation of cell force generation in living tissues and organs presents an exceptional challenge. The cellular microenvironment presents diverse biochemical and biophysical cues that dynamically modulate cell behavior. The complexity of these cues is amplified during processes such as morphogenesis, aging, and disease progression, which produce dramatic microenvironmental changes that enhance spatiotemporal heterogeneity. While numerous engineered models have been established to probe cellular responses to specific microenvironmental cues, a tremendous amount of work remains to be done to faithfully recapitulate tissue and organ complexity. Notably, countless biochemical and biophysical cues are present in the same tissue, which may obscure our understanding of cell behaviors when these cues are studied independently. In this section, we highlight recent advances in 3D multicellular spheroid models and organs-on-chips technology and discuss how these systems can be exploited to investigate traction force generation within complex microenvironments.

### Measuring Traction Force of 3D Multicellular Culture Models

Organs and tissues are highly dynamic, exhibiting spatiotemporal changes in the physical microenvironment and at the cellular level in the form of cell plasticity and heterogeneity. Due to computational and technological challenges, cell force generation remains understudied in the context of complex, multicellular tissues. Collectively, cells within spheroids and organoids can exert forces onto the surrounding ECM to facilitate physiological functions, such as collective cell migration, ECM remodeling, and tissue patterning (32, 103). By exerting traction forces on the ECM, it has been found that spheroids, in contrast to single cells, can more effectively align matrix fibers in radial directions, stiffen the matrix, and generate a high degree of ECM spatial heterogeneity, reminiscent of ECM changes observed in the tumor microenvironment (104).

In contrast to single-cell TFM, measuring the traction force of multicellular spheroids requires larger imaging volumes and the establishment of advanced methods that account for the spatial heterogeneity and dynamics of the ECM. The simplest approach to study spheroid-generated traction force is to measure the matrix deformation (86, 97, 103, 105). By leveraging the previously established TPT algorithm, Leggett et al. (86) established a method, called displacement array of rendered tractions, to track the displacements generated by multicellular clusters in composite SF–collagen hydrogels. Uniquely, their method enabled the visualization and analysis of heterogeneous displacements with high spatiotemporal resolution. Recent work established a reverse-engineered 3D tumor model of breast cancer heterogeneity and demonstrated how relatively rare invasive clonal cells direct tumor progression, which has likely implications in collective force generation in heterogeneous tumors (106). The Nelson group (107) established a robust technique for generating microfabricated tissues of defined geometry within a 3D matrix and tracked the embedded particles. Using this technique, they demonstrated the potential to estimate tissue-level traction forces. Other methods that have been successful in measuring matrix deformation include particle imaging velocimetry (103) and optical coherence microscopy (105), further improving data acquisition speed, duration, and imaging penetration distance. While these methods build on the success of the measurements of spheroid traction force–induced ECM deformation, it is recognized that the measurements of ECM deformation are limited because they require that the spheroids are embedded within the same ECM.

**Figure 6:**
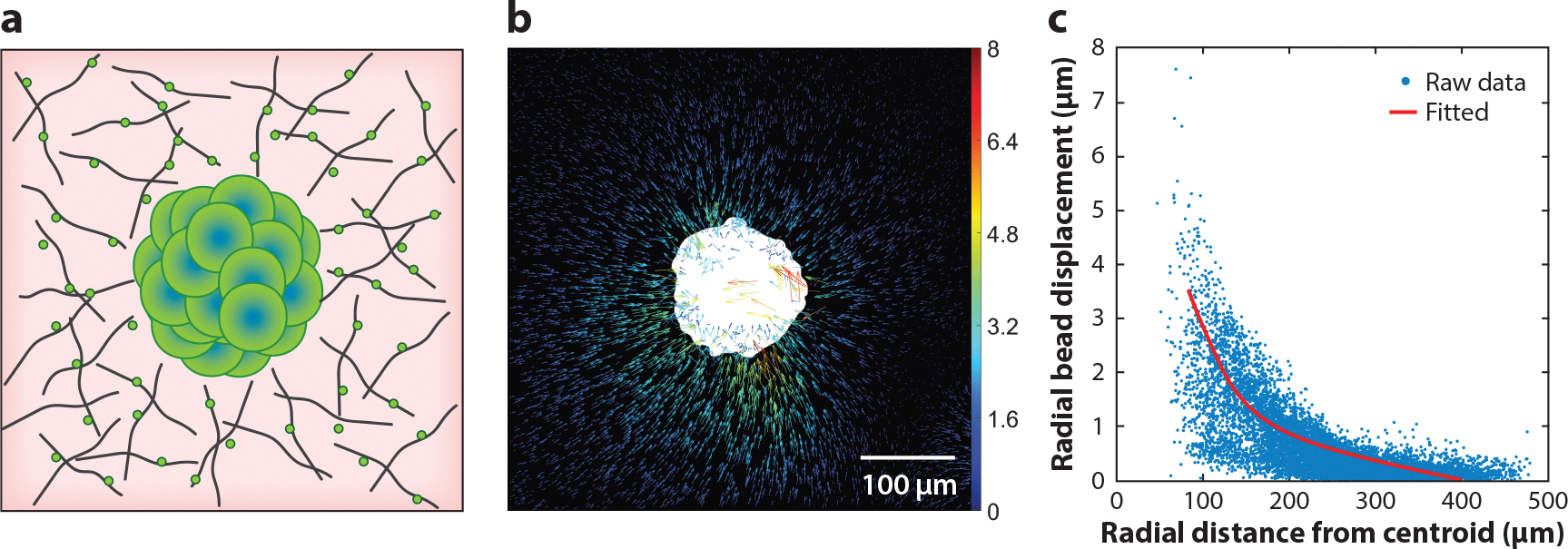
Traction force measurements for 3D tumor spheroids. (*a*) An illustration of a multicellular spheroid embedded in a collagen matrix. The green dots represent fluorescent markers used to assist in extracellular matrix (ECM) deformation measurements. (*b*) Experimentally measured ECM deformation field around a tumor spheroid (MDA-MB231 cells). Each arrow represents the ECM displacement measured by tracking one bead. (*c*) Radial bead displacement field. Each dot is a measured displacement from one bead. The solid red line is the result of least-squared fitting using a fibrous nonlinear elastic material model.

To take this a step further, traction force measurements of a tumor spheroid embedded within a 3D ECM have begun to appear (97). Taking advantage of the spherical symmetry of the spheroid, Mark et al. (97) deduced a scale-invariant relationship between the ECM deformation and the traction force. This calculation is now simplified and is in a public repository. In our labs, we have computed tumor spheroid–generated forces using a 3D TFM technique that was originally developed for single-cell studies (Figure 6). We note that the translation of ECM deformation to spheroid-generated traction force is significantly simplified due to the spherical symmetry of the spheroid. In addition to 3D TFM, soft particle–based techniques have been developed to explore local traction force, including a surface-treated PA microparticle (108) and a soft magnetic microrobotic method (109). In both cases, the deformation of a microsized particle of known material properties is used to measure the local traction force.

### Measuring Traction Force Under Controlled Biochemical Environments

Cell state and function are regulated not only by the physical environment but also by the constituents of the chemical environment; However, it is poorly understood how these synergistic cues dictate cell traction forces. Biochemical cues are presented in the form of bioactive molecules, such as growth factors, cytokines, ligands, and small molecules, which can be highly variable over space and time. For instance, morphogen gradients are often established in organisms over the course of development, enabling pattern formation in tissues. In mature tissues, analogous biochemical cues can aid in the establishment of cell polarity and directional migration, supporting processes such as wound healing and immune cell infiltration. Thus, it is critical to understand cell force generation in the context of both biochemical and mechanical cues in the microenvironment. Microfluidic platforms provide a unique opportunity for traction force measurement, in that they can recreate well-defined biochemical gradients in 3D and are compatible for optical imaging (110–112). Toward this goal, Jang et al. (113) designed a multichannel microfluidic device to produce a concentration of hepatocyte growth factor (HGF) through defined fluid flow. By performing TFM, they found that Madin-Darby canine kidney cell clusters display enhanced tension, traction forces, and migration in response to HGF in a concentration-, gradient-, and time-dependent manner, which correlated with changes in vinculin, E-cadherin, and F-actin localization and expression. Dou et al. (114) engineered a tunable microfluidic device that enabled the simultaneous generation of chemical gradients and stiffness profiles using PA hydrogels. They found that glioma cells presented with different morphologies, migration behaviors, and reactive oxygen species generation because of their exposure to these gradients. While these 2D culture models provide a starting point for tuning both biochemical and mechanical cues in a singular microfluidic device, these studies could be elevated by employing microfluidics housing 3D biological gels that have been generated for other applications (115–118).

### Measuring Traction Force Under Controlled Mechanical Microenvironments

Mechanosensation and mechanotransduction enable cells to adapt and respond to the physical aspects of the tissue microenvironment. While directed cell migration in response to local extracellular chemical gradients, or chemotaxis, has been recognized for more than a century and was first reported by W. Pfeffer in 1884 (119, 120), the role of physical microenvironmental cues in regulating dynamic cell behaviors is a recent development. Work by S. B. Carter in 1965 on substrates revealed that gradients of bound cues and adhesive sites direct cell migration, known as haptotaxis (121). Since then, numerous taxis phenomena have been established to describe the tendency of cells to migrate according to mechanical gradients in the cellular microenvironment, including substrate rigidity, or durotaxis (122); local, repeated anisotropy, or ratchetaxis (123); topography, or topotaxis (124); and cell-scale curvature, or curvotaxis (125). Furthermore, Mao et al. (126) recently coined the term diepafitaxis to describe the role of interfacial matrix stiffness and matrix–substrate adhesive strength.

Given the critical role of actomyosin contractility in cell migration, it is likely that these taxis phenomena are intricately linked to cell traction force. In support of this, for instance, the durotaxis studies by Lo et al. (122) were paired with measurements of cell force generation, revealing that fibroblasts exhibit stronger tractions on stiffer substrates. This example demonstrates that it is critical to consider how the local microenvironmental cues governing taxis phenomena may dictate traction force generation (122). Recently, Afthinos et al. (68) fabricated a microfluidic device termed a hydrogel-encapsulated microchannel array to study force generation during confined cell migration as a function of microenvironmental stiffness. 3D traction force measurements were made by tracking the displacement of fluorescent nanobeads within compliant PA-gel microchannels of known Young’s modulus, revealing that normal and shear traction forces, and total stress, exerted by cells during confined migration increased with microchannel stiffness (68).

## FUTURE OUTLOOK

Taken together, recent technological advances in experimental methods and computation have expanded the use of TFM from 2D to 3D microenvironments and from single-cell to tissue models, tremendously enhancing the physiological relevance of traction force measurements. However, several key challenges remain to be addressed in future work.

Current 3D TFM considers the reciprocal interactions between cells and ECMs through the fitting of theoretical data to those from experiments (17). The linkage of traction force measurements to molecular mechanisms of cell force generation would greatly advance our understanding of cell and tissue mechanobiology. We anticipate a future theoretical model that can directly address this cross talk between cells and ECMs through the modeling of both the mechanics of the actin–myosin network within the cell and the biopolymer network outside the cell, mechanically linked by the cellular adhesion complex. This multiscale model will have the potential to elucidate our understanding of how cytoskeletal molecules directly regulate ECM architecture, and vice versa, and dissect the roles of each molecule such as focal adhesion kinase and actin. We envisage the investigation of the correlation between integrin-specific engagement, RhoA expression, and cytoskeletal dynamics with cell traction force in real time. As a recent example, the development of molecular sensors (127, 128) could be used in conjunction with the multiscale models. To unveil the dynamics of RhoA during traction force generation, 3D TFM has also been multiplexed with Förster resonance energy transfer imaging of a RhoA biosensor (61, 129). It is also worth noting that to fully recapitulate the native environment of the ECM, one needs to develop a model in which all ECM components are considered. This entails profiling of the specific environment in which the cell resides. For example, the physiological relevance of culturing glioblastoma cells in a type I collagen gel is somewhat questionable, since the brain microenvironment consists mostly of hyaluronic acid. Nevertheless, most of the traction force measurements have been performed in such environments. While the mechanical responses of collagen gels have been studied extensively in recent years, a comparable mechanistic understanding of other biological gels remains nascent.

Although 3D biological gels can mimic some elements of the microenvironment in vivo, their material properties can vary over space and time due to dynamic remodeling of the ECM. Traction forces and cell/tissue stiffnesses are two fundamental features of mechanobiology. Even so, it has been challenging for researchers to quantify both 3D traction force and stiffness in the same location of a biological sample. Notably, little work has measured traction force in live tissues, and it remains especially challenging to do so while also characterizing physical properties of the ECM. Mohagheghian et al. (109) created a magnetic microrobot to quantify stiffness and forces of tumor colonies and embryos. With the magnetic microrobot, the researchers characterized the magnetic microrobot probe and quantified cancer cell colony modulus by the microrobotic probe and 3D traction forces. The stiffness and traction forces of zebrafish embryos were also quantified. These experiments revealed that cell-generated traction forces are important in regulating early embryogenic development and that different forces (normal versus shear) may be at play across species. Separately, they found that while the modulus of tumor-repopulating cell colonies varied with 3D substrate elasticity, their traction forces did not. This finding conflicts with behaviors observed in normal single cells but is consistent with those observed in vivo for the multilayer epithelia of mouse embryos, suggesting that a mechanical memory may dominate traction forces in these contexts (109). In an effort to understand traction force in the context of cellular-scale spatially resolved measurements, Lin et al. (51) developed a light-sheet photonic-force optical coherence elastography device for high-throughput quantitative 3D micromechanical imaging. This device was used to image fibrous collagen matrices with varying microarchitectures and demonstrated the potential for measuring micromechanical heterogeneities within these spatially heterogeneous matrices. Pairing this technology with live imaging could provide access to 4D characterization of matrix micromechanics with spatiotemporal variability, representing a significant advancement for the field of mechanobiology.

We anticipate the advent of novel 3D engineered culture models that controllably recapitulate such spatiotemporal heterogeneity and provide additional physiological cues, which could be paired with traction force measurements. For instance, to mimic spatial heterogeneity of the ECM, ongoing work has generated anisotropic matrices with stiffness gradients and local differences in architecture. Stimuli-responsive hydrogels could be leveraged to reveal traction forces associated with temporal changes in the ECM. The generation of matrices with varied ECM cross-linking and degradability would aid in our understanding of cell force generation in the context of matrix remodeling. These tunable ECM features are challenging to incorporate into biological gels, and, thus, these features have primarily been generated with synthetic hydrogel systems, likely obscuring critical insights into cell biomechanics that occur in vivo (130). Further, such sophisticated 3D culture systems present additional challenges for 3D TFM, which will need to evolve accordingly to be compatible with the introduction of these new experimental platforms. Moreover, for simplicity, much work with TFM in biological gels to date has focused on single cells; however, numerous engineered models have been established that incorporate tissue-like structures, such as organoids, cocultures, spheroids, and vascular networks (131). Overall, to advance our understanding of physiologically relevant tissue mechanics, it is critical to examine cell traction forces in these complex microenvironments.

**Figure 7:**
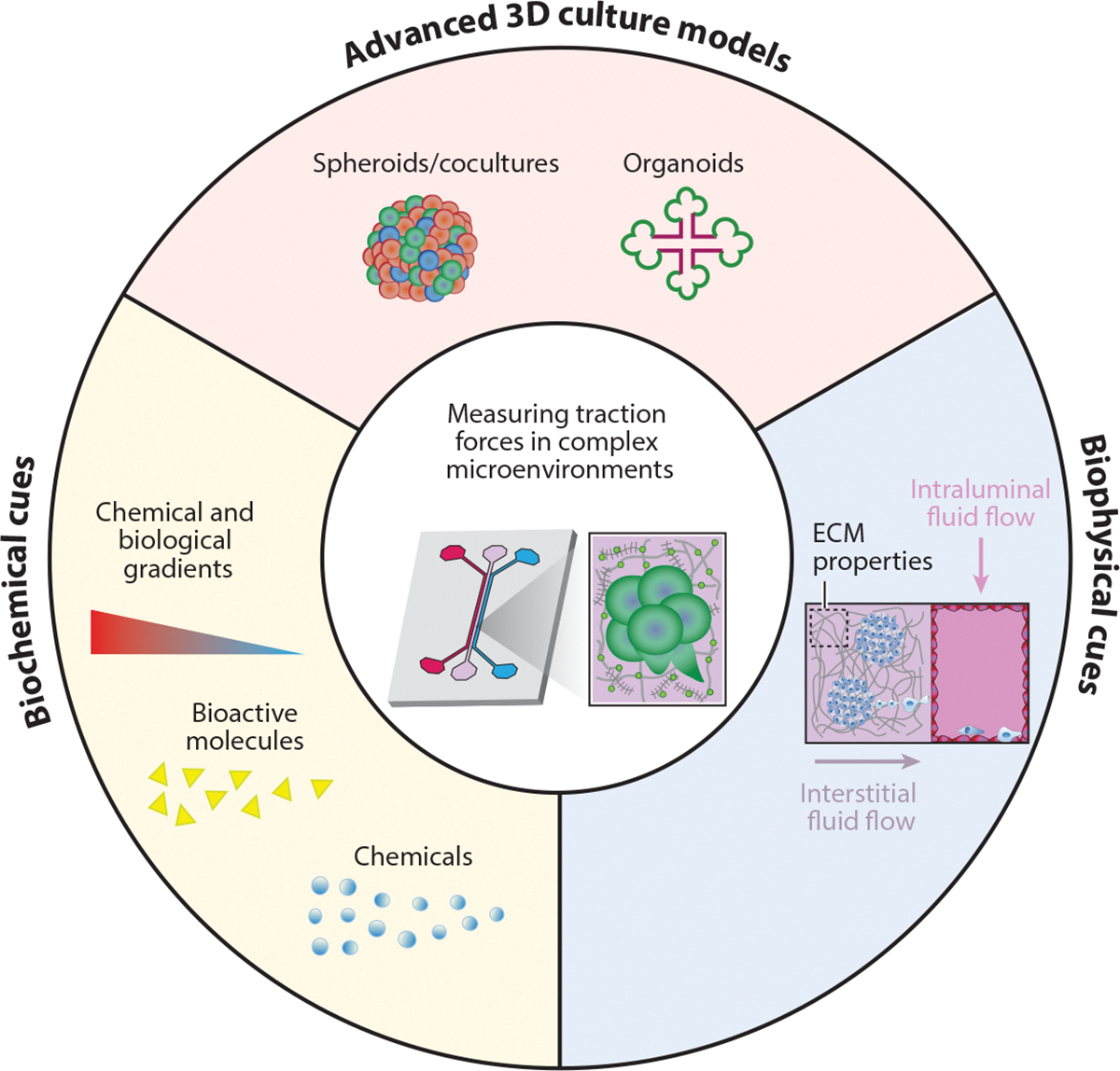
Overview of the diverse cellular and microenvironmental cues that could be uniquely combined in 3D engineered models for advanced traction force measurements. Bioengineered 3D culture models could incorporate multicellular structures (*top*), biochemical gradients (*left*), and fluid flows within biological gels with tunable extracellular matrix (ECM) properties (*right*) to interrogate cell traction force in physiologically relevant microenvironments.

Physiologically relevant fluid flows and biochemical gradients likely have important implications for cell force generation; however, these remain poorly understood. Recent advances in microfluidic devices have generated organ-on-a-chip systems that could be combined with traction force measurements. For instance, Dash et al. (132) developed a logarithmic device to generate a wide array of fluid shear stress (FSS), similar to what cells may experience in vivo. Cervical cancer cells (HeLa) cultured in the device displayed an increase in cancer cell stemness and drug resistance as FSS increased in magnitude. Mao et al. (133) developed a microfluidic device that consists of a gradient concentration generator that flows out to 10 blood vessels, creating 10 different Y-27632 (ROCK inhibitor) concentrations. Results demonstrated that the device was capable of mimicking blood vessel occlusion to measure how integrin tension was affected by low flow rates using blood platelets. Lee et al. (134) developed a multilayer microfluidic device (called MμLTI-Flow) consisting of (*a*) a tissue layer of parallel blood and lymphatic vessels embedded in collagen hydrogels, (*b*) a pressure regulatory layer, and (*c*) a permeable polycarbonate membrane separating the first two layers. The device design enables the independent control of luminal fluid flow, interstitial fluid flow, and interstitial fluid pressure with magnitudes observed in vivo (134). These advanced culture models could aid in the generation of dynamic and physiologically relevant microenvironments with which to study cell traction force generation over time. We envisage that these devices could be leveraged for the continuous monitoring of omics changes. As such, pairing these measurements with TFM could provide insight into the cross talk between the mechanome and other critical biological functions (Figure 7).

In addition to adapting to these advanced culture models, TFM will need to be optimized to better handle big data generated by high-throughput screening and longitudinal measurements. This is not a trivial task; instead, it would demand increased speed for imaging and measurements with higher spatiotemporal resolution. Further, assumptions used in computational models may limit the choice of materials used experimentally or lead to lower accuracy of measurements, which could be improved in the future. Critically, to benefit the widespread mechanobiology community, TFM platforms should be made easily accessible to nonexperts. This could be addressed by publishing protocols that incorporate paired experimental methods with TFM measurements. For instance, these should include detailed descriptions of the methods used to generate cell-laden biological gels, conduct TFM imaging, and perform subsequent analysis. In addition, applied computational models must be presented in a clear and user-friendly manner. This could be achieved by providing open-source low-code platforms or, ideally, no-code platforms with intuitive interfaces. Moreover, this could be enhanced with the inclusion of sample videos and paired datasets for users to test run the codes. While these approaches could permit widespread use of these platforms, a major barrier to their implementation is the significant increase in the time and effort that would need to be spent by developers.

Overall, TFM represents a powerful tool for understanding cellular force generation and corresponding biological consequences. The integration of advanced engineered tissue models, mechanical measurements, and the molecular underpinnings of traction force could illuminate these key behaviors in the context of the dynamic cell microenvironment and spatiotemporal heterogeneity. Achieving this exciting goal will require a highly interdisciplinary effort between engineers, biologists, physicists, and computer scientists. These models will provide critical insight into the role of cell force generation in physiologically relevant healthy and diseased states.

## Figures and Tables

**Table 1:** Summary of force measurement techniques

Method	Advantages	Limitations	ECM	Magnitude of force	Reference(s)
2D TFM	Does not require image reconstruction Materials are often isotropic	Does not recapitulate the physiological dimensionality Only lateral forces can be measured Applies only to linear elastic materials	Polyacrylamide PEG Silicone	nN to μN	91, 135–139
2.5D TFM	Can measure both lateral and normal forces Materials are often isotropic	Does not recapitulate the physiological dimensionality Applies only to linear elastic materials	Polyacrylamide (coated with collagen type I) PEG Silicone	nN to μN	69, 71, 140–147
3D TFM	Gels can be made to recapitulate the physiological environment Captures the adaptability of cell-ECM interaction Able to resolve forces from complex cellular features (e.g., protrusions)	Requires image reconstruction and particle tracking in 3D Material properties of some biological gels are not well understood Computationally expensive	Collagen type I Collagen type I-hyaluronic acid Collagen type I-fibrin	nN to μN	17,70, 73, 82, 96–98, 148
Micropillar assays	Enables force measurements at specific focal adhesions Pillar geometry is highly reproducible Force calculation is straightforward	Does not recapitulate the physiological dimensionality Applies only to linear elastic materials Not compatible with certain cell types (e.g., T cells)	PDMS	pN to nN	149, 150
Microparticle TFM	Reveals forces between immune cell-target interactions Tunable target stiffness	Requires prior knowledge of mechanical properties of target Limited to linear elastic target Requires image reconstruction	Deformable poly(AAm-co-AAc)	nN	151
Monolayer stress microscopy	Provides insights at the tissue level	Resolution of force within the whole layer is limited by the field of view	Polyacrylamide	nN to μN	152–154

Abbreviations: ECM, extracellular matrix; PEG, poly(ethylene glycol); PDMS, poly(dimethylsiloxane); poly(AAm-co-AAc), poly(acrylamide-co-acrylic acid); TFM, traction force microscopy.
